# Simultaneous spectrophotometric determination of paracetamol, phenylephrine and chlropheniramine in pharmaceuticals using chemometric approaches

**Published:** 2010

**Authors:** M.R. Khoshayand, H. Abdollahi, A. Ghaffari, M. Shariatpanahi, H. Farzanegan

**Affiliations:** 1 Department of Drug and Food Control, Faculty of Pharmacy and Pharmaceutical Sciences Research Center, Tehran University of Medical Sciences, Tehran; 2 Department of Chemistry, Institute for Advanced Studies in Basic Sciences, Zanjan; 3 Chemidarou Pharmaceutical Company, Tehran, Iran

**Keywords:** Principal components regression (PCR), Partial least squares regressions (PLS1, PLS2)

## Abstract

**Background and the purpose of the study:**

The linear multivariate calibration models such as principal components regression (PCR) and partial least squares regressions (PLS1 and PLS2) due to the mathematical simplicity and physical or chemical interpretability are sufficient and generally preferred method for analysis of multicomponent drugs. In this study, simultaneous determination of paracetamol, phenylephrine and chlorpheniramine in pharmaceuticals using chemometric methods and UV spectrophotometry is reported as a simple alternative technique.

**Material and methods:**

Principal components regression (PCR) and partial least squares regressions (PLS1 and PLS2) were used for chemometric analyses of data obtained from the spectra of paracetamol, phenylephrine and chlorpheniramine between wavelengths of 200 to 400 nm at several concentrations within their linear ranges. The analytical performance of these chemometric methods were characterized by relative prediction errors and recoveries (%) and compared with each other.

**Results:**

PCR, PLS1 and PLS2 were successfully applied to a tablet formulation, with no interference from excipients as indicated by the recovery. However, the PLS1 shows better results due to its flexibility and mathematical principals.

**Conclusion:**

The proposed methods are simple and rapid requiring no separation step, and can be easily used as an alternative in the quality control of drugs.

## INTRODUCTION

The combination of the paracetamol (PCT), phenylephrine (PHEN) and chlorpheniramine (CHL) is frequently used as active ingredients in cold medications due to their analgesic, antipyretic, decongestant and antihistaminic activities (1).

While official methods (2) are available for determination of the each of the above compounds alone in the formulations, the most prominent method for simultaneous determination of PCT, PHEN and CHL is the HPLC (3). However, basic characteristics of PCT, PHEN and CHL make them suitable for reaction with the stationary phase of reverse phase HPLC and accordingly results in peak asymmetry and low separation efficiency (4). Marin et al reviewed the analytical methods published for determination of these drugs in mixtures (5). Although, HPLC and capillary electrophoresis (6), allowed the separation and assay of these drugs, they are costly and time consuming. Additionally the United States pharmacopoeia has suggested the reduction in amount of reagents and materials which are routinely used in HPLC assays that have the potential to cause harm to human health and environment. Therefore, spectrophotometry as a simple, robust, quick and low cost method may be a good alternative if it is combined with multivariate calibration methods for determination of a complex mixture in pharmaceutical quality control laboratories.

Application of multivariate calibration techniques on overlapping data offers complex system resolution without preliminary separation steps required for classical spectrophotometric methods.

Principal components regression (PCR) and partial least squares regressions (PLS1 and PLS2) (7) due to the mathematical simplicity and physical or chemical interpretability (8) have been successfully used in quantitative determination of many pharmaceutical preparations (9–14) and seems to be the best known chemometric algorithms in multicomponent pharmaceutical systems (15).

In this study, a simple, rapid and inexpensive method for simultaneous spectrophotometric determination of PCT, PHEN and CHL is proposed. Due to the heavily overlapped data, this method processed by multivariate calibration techniques including PCR and two versions of the PLS algorithm: PLS1 and PLS2 and determined their concentration, both in their mixtures and a tablet formulation. To the best of our knowledge, there is no other previous report for the simultaneous spectrophotometric determination of these drugs in synthetic or a pharmaceutical formulation by chemometric methods. The predictive ability of multivariate calibration methods, including PCR, PLS1 and PLS2, were investigated and successfully compared with each other.

## MATERIAL AND METHODS

### 

#### Material

##### Reagent and solutions

All reagents used were analytical grade (Merck and Fluka) and used directly without further purification. PCT, PHEN and CHL were kindly donated by Rouz Darou. Pharmaceutical company. Triple distilled water was used to prepare buffer and reagent solutions. Stock solutions of Paracetamol, phenylephrine and chlorpheniramine in concentration of 1000 µg ml-1 were prepared in 100 ml volumetric flasks, by dissolving 100 mg of each compound in methanol:0.1 M HCl (3:1). All solutions were prepared fresh daily.

##### Pharmaceutical preparation

A commercial Adult cold® tables manufactured by Rouz Darou laboratories (Tehran-Iran) batch no: D.259) was procured from local drug stores and assayed. The nominal quantity of active ingredients in each tablet was as follows: paracetamol BP (325 mg), phenylephrine HCl (5 mg) and chlorpheniramine maleate (2 mg).

#### Methods

##### Apparatus and software

A dual-beam GBC Cintra 101 spectrophotometer, with 1 cm quartz cells, a scan rate of 1000 nm min^-1^ and the slit width of 2 nm was used for collection of the digitized UV-VIS absorbency spectra. The UV spectra of mixtures were recorded over the wavelength 200-400 nm with one data point per nanometer. They were saved in ASCII format and transferred to a Pentium (IV) microcomputer for running the programs. All spectral measurements were performed using blank solution as a reference. Measurements of pH were made with a Metrohm 691 pH-meter using a combined glass electrode. PCR, PLS1 and PLS2 methods were used for chemometric analyses of data. For all calculations Matlab for windows (version 7.0) (16) was used. PCA, PLS1 and PLS2 methods were carried out with the PLS-Toolbox (17).

##### Standard solutions

Working standard solutions were prepared daily in the linear calibration range by diluting the stock solutions for each drug. Standard solutions were prepared in two sets as follows: The calibration set and prediction sets containing 20 and 10 solutions respectively. To a series of 10 ml volumetric flasks, aliquots of PCT, PHEN and CHL solutions, containing appropriate amount of these drugs in the range of calibrations, were added and then the solutions were diluted to 10 ml with methanol: 0.1 M HCl (3:1). UV spectra of the mixtures were recorded in the wavelength range of 200-400 nm versus a solvent blank, and digitized absorbance was recorded at 1 nm intervals.

##### Sample preparation

Twenty tablets were powdered in a mortar and mixed. An amount of powder equivalent to one tablet was accurately weighted and transferred into a volumetric flask using methanol: 0.1 M HCl (3:1) and dissolved by mechanical shaking for 30 min. The solution was filtered into a 100 ml volumetric flask through the Whatman No. 42 filter paper, diluted with same solvent, and then adjusted to the volume of 100 ml. This stock solution furnished suitable working sample solutions for UV measurements and analyzed by proposed chemometrics methods.

## RESULTS AND DISCUSSION

### 

#### Individual calibration

**Figure 1:**
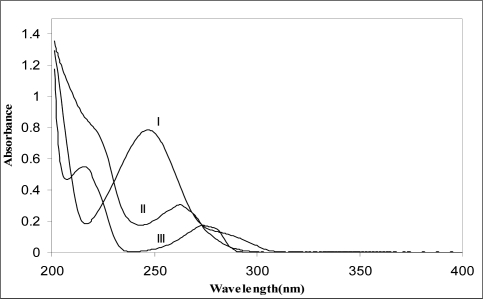
Absorbance spectra of: (I) 10 µg ml^-1^ Paracetamol; (II)10 µg ml^-1^ Phenylephrine; (III) 10 µg ml^-1^ Chlorpheniramine in methanol: 0.1 M HCl (3:1).

The UV absorption spectra for PCT, PHEN and CHL in the standard solutions, which were recorded between 200 and 400 nm, are shown in Fig. 1.

The calibration curves of these drugs in the range of 1-17 µg ml-1 for PCT, 1-20 µg ml-1 for PHEN and 1-20 µg ml-1 for CHL, were drawn with several points as absorbance versus drugs concentrations and statistically evaluated by linear regression. The intercepts on the ordinates were negligible in the calibration lines. Limits of detection was calculated as 3*s*_*0*_ per slope (where *s*_*0*_ is the standard deviation of the intercept on the ordinate), were 0.49 µg ml-1 for PCT, 0.57 µg m1-1 for PHEN, and 0.65 µg m1-1 for CHL, respectively.

#### Multivariate methods

Calibration matrix construction is the first step in simultaneous determination of ternary mixture of PCT, PHEN and CHL by multivariate calibration methods.

Twenty ternary mixtures were selected by random design as the calibration set to fulfill the non-correlated concentration matrix (Table 1). The correlation coefficients between the vectors were considered as the criteria to minimize the relationship between the drug concentrations. The calibration model in each chemometric method was validated with 10 synthetic mixtures set. The predictive abilities of PCR, PLS1 and PLS2 were examined for simultaneous determination of PCT, PHEN and CHL in sample mixtures.

**Table 1:** Composition of the calibration set for applying PCR, PLS1 and PLS2 methods.

Number of calibration sample	PCT (µgml-1)	PHEN (µgml-1)	CHL (µgml-1)
1	20.0	20.0	6.0
2	15.0	12.0	16.0
3	18.0	1.0	1.0
4	19.0	17.0	1.0
5	18.0	12.0	15.0
6	10.0	2.0	14.0
7	19.0	12.0	10.0
8	7.0	8.0	9.0
9	2.0	17.0	6.0
10	14.0	2.0	17.0
11	12.0	1.0	10.0
12	3.0	7.0	2.0
13	17.0	13.0	2.0
14	4.0	17.0	18.0
15	1.0	12.0	18.0
16	19.0	14.0	15.0
17	4.0	14.0	4.0
18	14.0	15.0	2.0
19	15.0	11.0	15.0
20	10.0	3.0	11.0

**Figure 2:**
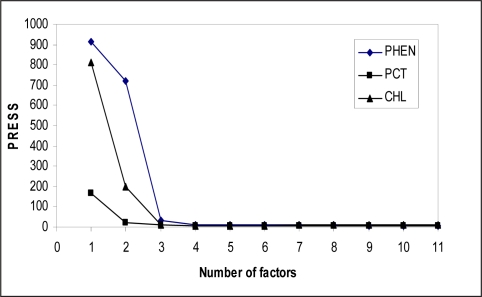
Plot of PRESS against the number of factors for PCT, PHEN and CHL.

Before construction of the model, selection of the optimum number of factors for PCR and PLS multivariate calibration techniques are very important. In this study the cross validation method, leaving one sample out, was used to select the optimum number of factors. For each prediction set of PCT, PHEN and CHL, the prediction residual error sum of squares (PRESS) was calculated as an indicator for adequacy of models. Fig. 2 shows the plots of PRESS versus the number of factors for PCR, PLS1 and PLS2 techniques. In order to find the smallest model with fewest numbers of factors, the **F** statistics was used to carry out the significant determination. The optimal numbers of factors for PCT, PHEN and CHL in each model were as 2, 3 and 3, respectively.

In this work, 10 synthetic test samples were analyzed with the proposed methods. The prediction results are given in Tables 2–4. The prediction error of a single component in the mixture was calculated as the relative standard error (R.S.E) of the prediction concentrations (18). Tables 2–4, also show reasonable single and total relative standard error for such a system.

**Table 2:** Composition of synthetic samples, their predictions by PCR model and statistical parameters for the system.

Synthetic (µgml-1)	Prediction(µgml-1)				Recovery (%)			

PCT	PHEN	CHL	PCT	PHEN	CHL	PCT	PHEN	CHL
18.0	10.0	16.0	17.8	10.3	14.7	98.9	103.0	91.9
17.0	1.0	11.0	17.8	0.98	11.01	104.7	98.0	100.1
9.0	20.0	16.0	8.3	19.7	15.8	92.2	98.5	98.8
9.0	13.0	6.0	8.8	13.1	5.2	97.8	100.8	86.7
16.0	4.0	19.0	15.8	4.1	17.8	98.8	102.5	93.7
2.0	10.0	11.0	2.3	9.7	10.7	115.0	97.0	97.3
10.0	13.0	20.0	8.9	13.3	19.2	89.0	102.3	96.0
4.0	1.0	15.0	4.1	1.1	15.1	102.5	101.0	100.7
5.0	7.0	11.0	4.8	6.3	11.5	96.0	90.0	104.5
19.0	13.0	4.0	18.8	13.8	3.8	98.9	106.2	95.0
Mean recovery						99.4	99.9	96.5
R.S.E. (%) single						4.14	3.58	5.03
R.S.E. (%) total							4.41	

**Table 3:** Composition of synthetic samples, their predictions by PLS1 model and statistical parameters for the system.

Synthetic (µgml-1)	Prediction (µgml-1)				Recovery (%)			

PCT	PHEN	CHL	PCT	PHEN	CHL	PCT	PHEN	CHL
18.0	10.0	16.0	18.1	10.1	15.7	100.6	101.0	98.1
17.0	1.0	11.0	17.9	1.0	11.2	105.3	100.0	101.8
9.0	20.0	16.0	8.6	19.8	16.3	95.6	99.0	101.9
9.0	13.0	6.0	8.8	13.0	5.6	97.8	100.0	93.3
16.0	4.0	19.0	15.9	4.1	17.9	99.4	102.5	94.2
2.0	10.0	11.0	2.3	9.8	10.9	115.0	98.0	99.1
10.0	13.0	20.0	9.8	13.2	19.1	98.0	101.5	95.5
4.0	1.0	15.0	4.1	0.99	15.6	102.5	99.0	104.0
5.0	7.0	11.0	4.9	6.8	11.2	98.0	97.1	101.8
19.0	13.0	4.0	18.9	13.5	3.9	99.5	103.8	97.5
Mean recovery						101.2	100.2	98.7
R.S.E. (%) single						2.78	1.91	3.84
R.S.E. (%) total							3.10	

**Table 4:** Composition of synthetic samples, their predictions by PLS2 model and statistical parameters for the system.

Synthetic (µgml-1)	Prediction (µgml-1)				Recovery (%)			

PCT	PHEN	CHL	PCT	PHEN	CHL	PCT	PHEN	CHL
18.0	10.0	16.0	18.2	9.9	15.5	101.1	99.0	96.9
17.0	1.0	11.0	17.5	1.07	11.3	102.9	107.0	102.7
9.0	20.0	16.0	9.1	20.1	16.1	101.1	100.5	100.6
9.0	13.0	6.0	8.3	12.8	6.6	92.2	98.5	110.0
16.0	4.0	19.0	16.3	3.8	18.3	101.9	95.0	96.3
2.0	10.0	11.0	1.9	10.3	11.1	95.0	103.0	100.9
10.0	13.0	20.0	9.2	12.99	18.4	92.0	99.9	92.0
4.0	1.0	15.0	3.8	0.99	15.3	95.0	99.0	102.0
5.0	7.0	11.0	5.1	6.5	11.4	102.0	92.9	103.6
19.0	13.0	4.0	19.1	13.8	3.9	100.5	106.2	97.5
Mean recovery						98.4	100.1	100.3
R.S.E. (%) single						3.22	3.08	4.59
R.S.E. (%) total							3.81	

#### Applications

**Figure 3:**
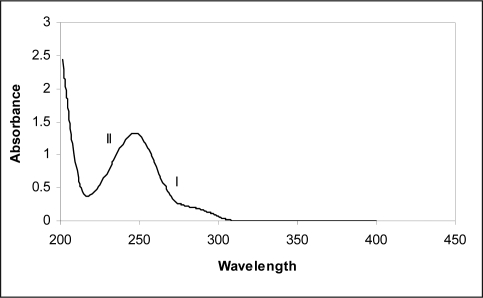
Absorbance spectra of: (I) Mixture of PCT, PHEN and CHL; (II) Commercial formulation (Adult cold) in methanol: 0.1 M HCl (3:1).

The proposed methods were applied to several real samples for determination of these drugs in a tablet formulation. Five replicate measurements were made and the results are shown in Table 5. The good agreement between these results and the label claims indicates the successful application of the proposed procedure for the simultaneous determination of PCT, PHEN and CHL in real sample. To check the validity of the proposed method, standard addition method was implemented. It was found that the amount of these drugs did not change by adding the known amounts of PCT, PHEN and CHL (data not shown). Moreover, a comparison between the spectra obtained from the mixture of PCT, PHEN and CHL in standard and drug formulation solutions showed complete similar patterns (Fig. 3). Therefore, the excipient ingredients in commercial preparation did not interfere with the measurement of PCT, PHEN and CHL in the pharmaceutical formulation.

**Table 5:** Assayed results of simultaneous determination of PCT, PHEN and CHL in Adult cold tablets by the proposed methods.

	PCT			PHEN			CHL		

	PCR	PLS1	PLS2	PCR	PLS1	PLS2	PCR	PLS1	PLS2
Sample 1 (mg)	324.0	323.2	323.9	5.01	5.03	5.05	1.99	2.02	1.99
Sample 2 (mg)	323.5	325.6	324.0	5.02	5.01	5.04	1.98	2.01	2.01
Sample 3 (mg)	325.5	324.8	326.3	4.99	5.02	4.99	1.96	1.99	2.01
Sample 4 (mg)	323.7	326.1	326.1	4.98	4.98	4.97	2.01	1.97	1.97
Sample 5 (mg)	325.5	325.6	324.2	4.98	4.97	5.08	2.01	1.98	1.99
Amount on the label (mg)	325.0	325.0	325.0	5.00	5.00	5.00	2.00	2.00	2.00
Mean% recovery	99.8	100.0	99.9	99.9	100.0	100.5	99.5	99.7	99.7
SD% recovery	0.30	0.35	0.37	0.35	0.52	0.90	1.06	1.04	0.84

## CONCLUSIONS

This study established the feasibility of simultaneous determination of PCT, PHEN and CHL in synthetic and pharmaceutical preparations by a simple and rapid method without any time-consummation for sample preparation. Generally, it was found that all methods are accurate to model the considered system for determination of these drugs. However, the superiority of PLS1 over other applied multivariate methods is its flexibility and mathematical point of view. High percentage of recovery shows that the methods are free from interference of the excipients used in the commercial formulation. Results also reveal that the developed methods can be applied for a routine analysis and quantitative control of mixtures and commercial preparations containing these three drugs.
